# Individual Case Analysis of Postmortem Interval Time on Brain Tissue Preservation

**DOI:** 10.1371/journal.pone.0151615

**Published:** 2016-03-16

**Authors:** Jeffrey A. Blair, Chunyu Wang, Damarys Hernandez, Sandra L. Siedlak, Mark S. Rodgers, Rojan K. Achar, Lara M. Fahmy, Sandy L. Torres, Robert B. Petersen, Xiongwei Zhu, Gemma Casadesus, Hyoung-gon Lee

**Affiliations:** 1 Department of Biological Sciences and School of Biomedical Sciences, Kent State University, Kent, Ohio, United States of America; 2 Department of Pathology, Case Western Reserve University, Cleveland, Ohio, United States of America; 3 Department of Neurology, The Second Xiangya Hospital, Central South University, Changsha, Hunan, People's Republic of China; New York State Institute for Basic Research, UNITED STATES

## Abstract

At autopsy, the time that has elapsed since the time of death is routinely documented and noted as the postmortem interval (PMI). The PMI of human tissue samples is a parameter often reported in research studies and comparable PMI is preferred when comparing different populations, i.e., disease versus control patients. In theory, a short PMI may alleviate non-experimental protein denaturation, enzyme activity, and other chemical changes such as the pH, which could affect protein and nucleic acid integrity. Previous studies have compared PMI en masse by looking at many different individual cases each with one unique PMI, which may be affected by individual variance. To overcome this obstacle, in this study human hippocampal segments from the same individuals were sampled at different time points after autopsy creating a series of PMIs for each case. Frozen and fixed tissue was then examined by Western blot, RT-PCR, and immunohistochemistry to evaluate the effect of extended PMI on proteins, nucleic acids, and tissue morphology. In our results, immunostaining profiles for most proteins remained unchanged even after PMI of over 50 h, yet by Western blot distinctive degradation patterns were observed in different protein species. Finally, RNA integrity was lower after extended PMI; however, RNA preservation was variable among cases suggesting antemortem factors may play a larger role than PMI in protein and nucleic acid integrity.

## Introduction

In the field of neuropathology, one parameter that is variable and difficult to control is the time between death and the sampling of tissue for making a diagnosis. Most often, the body is refrigerated awaiting autopsy, yet the postmortem interval (PMI) can range from 1 h to well over 24 h. To make an accurate diagnosis, good tissue preservation for morphological analysis and antigenic preservation for immunohistochemical analysis is crucial. Often, DNA and RNA are also purified for either genetic analysis or research purposes. Changes that occur during this interval affect enzyme activity, nucleic acid integrity, oxidative modification, and protein integrity. During the time of pre-autopsy, while bodies are stored at 4°C, the medial sections of the brain cool more slowly and are consequently more affected by enzymes and biological processes than the lateral portions [1]. Recently, postmortem imaging studies that investigate potential *in vivo* imaging of ischemia and multiple sclerosis have begun to take into account PMI, showing a necessity to directly study its effect [2, 3]. A lengthy PMI has been shown to affect RNA integrity in microarray experiments [4], while apoptotic factors and pH have been found to be unrelated to PMI [5–7]. Phosphorylated residues in the mouse brain are dramatically affected by PMI, and the same study confirmed that while storing tissue at 4°C did not inhibit dephosphorylation, it did delay the decrease in total protein levels when compared to storage at room temperature [8]. In fact, GSK3 is rapidly dephosphorylated at its phospho-serine residues, with 95% dephosphorylation in as little as 10 min postmortem [9]. One study compared human brain biopsy tissue held for up to 4 h after sampling and found similar loss of phosphorylated tau within 1 h [10].

While suspected cases of neurodegenerative disease are often given high priority for rapid autopsy, tissue samples from non-affected control patients, while just as important for human tissue studies, are often not collected with the same rigor with respect to minimizing the time between death and autopsy. This is primarily due to the fact that the tissues are not the focus for determining cause of death. Indeed, while the effect of PMI is often debated, studies to date have not systematically tested the effects of time delay on individual patient samples. Most studies have only compared different cases or different animal models to evaluate the PMI variables. To overcome some of these experimental weaknesses, we sampled, fixed, and embedded human hippocampal tissue at multiple time points from individual cases. Using the initial time point as the control and the subsequent time points as the experimental sample, the PMI was an isolated variable within a single case. Tissue morphology, a variety of common antibodies used for immunohistochemistry or Western blot analysis, phosphorylation changes, and RNA integrity were examined in this study. With this experimental approach, the effect of PMI was analyzed without requiring a large sample population to control for physiological differences. The findings of this study are important for human tissue studies that use autopsy and archived tissue samples.

## Materials and Methods

**Fig 1:**
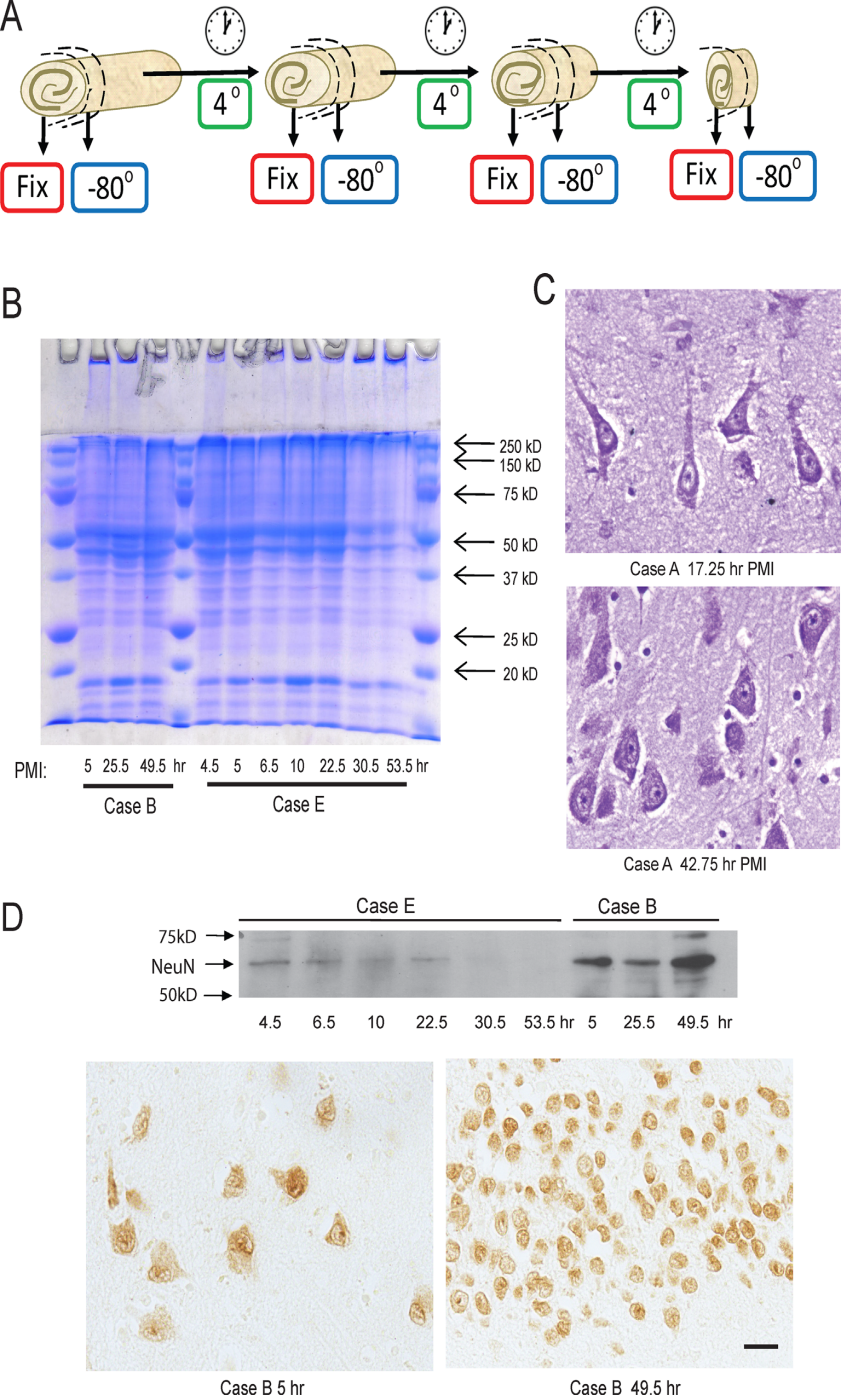
Tissue sampling protocol and tissue morphology with increased PMI. (A) The diagram shows how the hippocampus tissues were sampled at the various timepoints. The number of timepoints collected depended on the size of the original hippocampus sample. Coomassie blue stain of samples from the two cases with initially low PMI collected at different PMI times and frozen. Homogenates were made and equal amounts of protein run on the gel. Case #s refer to Table 1. (B) Case B shows no apparent loss of total protein load, while Case E shows overall weaker Coomassie stain at timepoints of 30 and 53 h. (C) Morphologically, minimal tissue or cellular changes are noted with increased PMI by Nissl stain. (D) Western blot analysis for NeuN finds there is no decrease of the NeuN band with PMI in Case B, but Case E shows both lower overall NeuN levels and a loss of NeuN at the longest PMI times. Immunostaining Case E shows that both pyramidal neurons and dentate gyrus neurons show the expected neuronal localization of NeuN at all PMI times. Scale bar = 20 μm.

Autopsy tissue was obtained from University Hospitals of Case Medical Center under an IRB protocol (03-00-26) approved by The University Hospitals of Case Medical Center Institutional Review Board which contains Waiver of Consent, Waiver of Assent and Parental Permission and Waiver of HIPAA Authorization. Large hippocampal sections were resected and stored in a closed plastic container at 4°C. At pre-determined experimental time points, a portion of the resected hippocampus was removed and either placed at -80°C or in 10% buffered formalin formaldehyde. The remaining tissue was returned to 4°C until the subsequent time point, at which point an identical fixative and sampling/storage procedures were used (Fig 1A). The size of the initial sample determined how many time points could be collected per case. For each sampling time, a cross section area approximately 2–4 mm thick of the hippocampus was collected, including readily identified dentate gyrus and cornus ammonus regions. The two cases with the shortest initial PMI (approximately 4 and 5 h) were used for RNA and protein analysis. The fixed tissue samples were paraffin embedded, sectioned at 6 μm with a microtome, and analyzed histochemically and immunohistochemically. Table 1 lists the case details and the PMIs studied for each case, and Fig 1 shows a schematic of tissue collection procedure.

**Table 1:** Cases used in the study and the tissue collection times for each case.

Case	Age	Gender	Neuropathological Diagnosis	Initial PMI (h)	Additional samples (fixed)	Additional samples (frozen)
A	42	M	No significant gross or histopathologic abnormality	17.25	+25.5 (42.75 h total)	NA
B	73	F	Alzheimer’s disease	5.00	+44.5 (49.5 h total)	+20.5 (25.5 h total)
			Braak VI			+44.5 (49.5 h total)
C	75	M	Severe atherosclerosis of	22.50	+ 4 (26.5 h total)	NA
			intracranial arteries		+19 (41.5 h total)	
D	77	M	Moderate atherosclerosis of intracranial vessels	23.00	+23 (46 h total)	NA
E	78	M	Alzheimer’s disease	4.50	+2 (6.5 h total)	+1 (5.5 h total)
			Braak VI		+26 (30.5 h total)	+2 (6.5 h total)
					+49 (53.5 h total)	+5.5 (10 h total)
						+18 (22.5 h total)
						+26 (30.5 h total)
						+49 (53.5 h total)

Paraffin sections were hydrated with two 10-min submersions in xylene followed by equilibration in a series of descending alcohol solutions also for 10 min each. Between the 95% and 70% alcohol solutions, the tissue sections were submersed for 30 min in a 3% H_2_O_2_ solution and after the final alcohol solution the sections were submersed in TBS for at least 10 min. The immunohistochemical analysis focused on a range of antibodies that are routinely used for neuropathological and normal anatomical data. Antibodies for collagen IV, SMI-34 (anti phosphoneurofilaments, Abcam), HNE (marker of lipid peroxidation, Alpha Diagnostic Internations), α-tubulin (Invitrogen, clone B-5-1-2), COX-1 (cytochrome oxidase 1, Molecular Probes), double stranded DNA (Millipore), NeuN (Millipore), phosphorylated tau Ser396/404 (PHF1, gift of Peter Davies), phosphorylated tau Ser202/Thr205 (AT8, Thermofisher), GFAP (astrocytic marker, Invitrogen), and rRNA (Millipore) were studied. Nissl stain (cresyl violet) was also performed.

For Western blot analysis, grey matter from the frozen samples from the two cases that had the shortest initial PMI were homogenized in 10X volume of lysis buffer (Cell Signaling Technologies) with protease inhibitors and phosphatase inhibitors added (Roche Diagnostics). Samples were centrifuged at 4°C, and the supernatants collected and aliquoted. For this experiment, the effect of storage time on protein stability was the experimental parameter, so to overcome any discrepancy in loading levels, BCA was used to determine the protein concentration, and 200 μl stock solutions at 1 mg/ml concentration were prepared from each sample supernatant. 10 μg or 30 μg of protein per well was resolved by electrophoresis on 10% SDS PAGs and the protein was transferred to immobilon membranes. After blocking with 10% dry milk, membranes were incubated with primary antibodies overnight followed by rinsing 5 times for 5 min each in TBS-Tween. An HRP conjugated secondary antibody was applied for 1 h, followed by rinsing again as above. Protein bands were detected with ECL reagent (Santa Cruz and Millipore). Coomassie blue staining of gels from the two shortest PMI cases was also performed. The primary antibodies used for detection on Western blots included α-tubulin (Invitrogen), β-actin (Millipore), GAPDH (Abcam), PHF1, AT8, NeuN, and tau-5 (Pierce). Quantification was made relative to highest value per antibody per case or relative to actin and a regression analysis was done to compare the relative levels with PMI time points.

To compare how PMI affects RNA stability, RNA was prepared from all PMI time points collected from the two cases with the shortest PMI, approximately 4 h, and compared to a mouse RNA standard, essentially 0 h PMI, prepared at the same time on an ethidium bromide stained gel. Quantitative RT-PCR (qRT-PCR) was used to evaluate two commonly measured housekeeping genes: GAPDH and β2-microglobulin (B2M), as previously described [11]. RNA purification was performed in duplicate for each case from the original frozen starting material. qRT-PCR reactions were performed only on the samples for which enough RNA was obtained. Both cases (Case B and Case E) were assayed at the same time for both RNA purification and qRT-PCR analysis.

## Results

### Protein integrity and tissue morphology

Tissue from individual patients sampled at different times after autopsy, were used to evaluate protein integrity and tissue morphology. Frozen tissue samples were homogenized, protein levels determined and 30 μg protein per well was resolved using 10% SDS-PAGE and the gel was stained with Coomassie blue. Fig 1B shows the results from two cases. After 24 h, some “smears’ were noticeable in the gel in Case B and weaker stained bands in Case E, but overall the proteins resolved were largely unchanged. The tissue samples fixed after the initial PMI and then up to an additional 49 h at 4°C exhibited no striking changes in neuronal morphology using routine Nissl stain (representative case shown in Fig 1C). Tissue samples contained both pyramidal neurons in the cornus ammonus regions and neurons in the dentate gyrus areas detected by NeuN (Fig 1D). By Western blot, NeuN levels were unchanged with PMI in Case B, but showed less staining in the samples with the longest time points in Case E (Fig 1D), correlating with the Coomassie staining pattern shown in Fig 1B.

### Effect of PMI on cytoskeletal proteins

**Fig 2:**
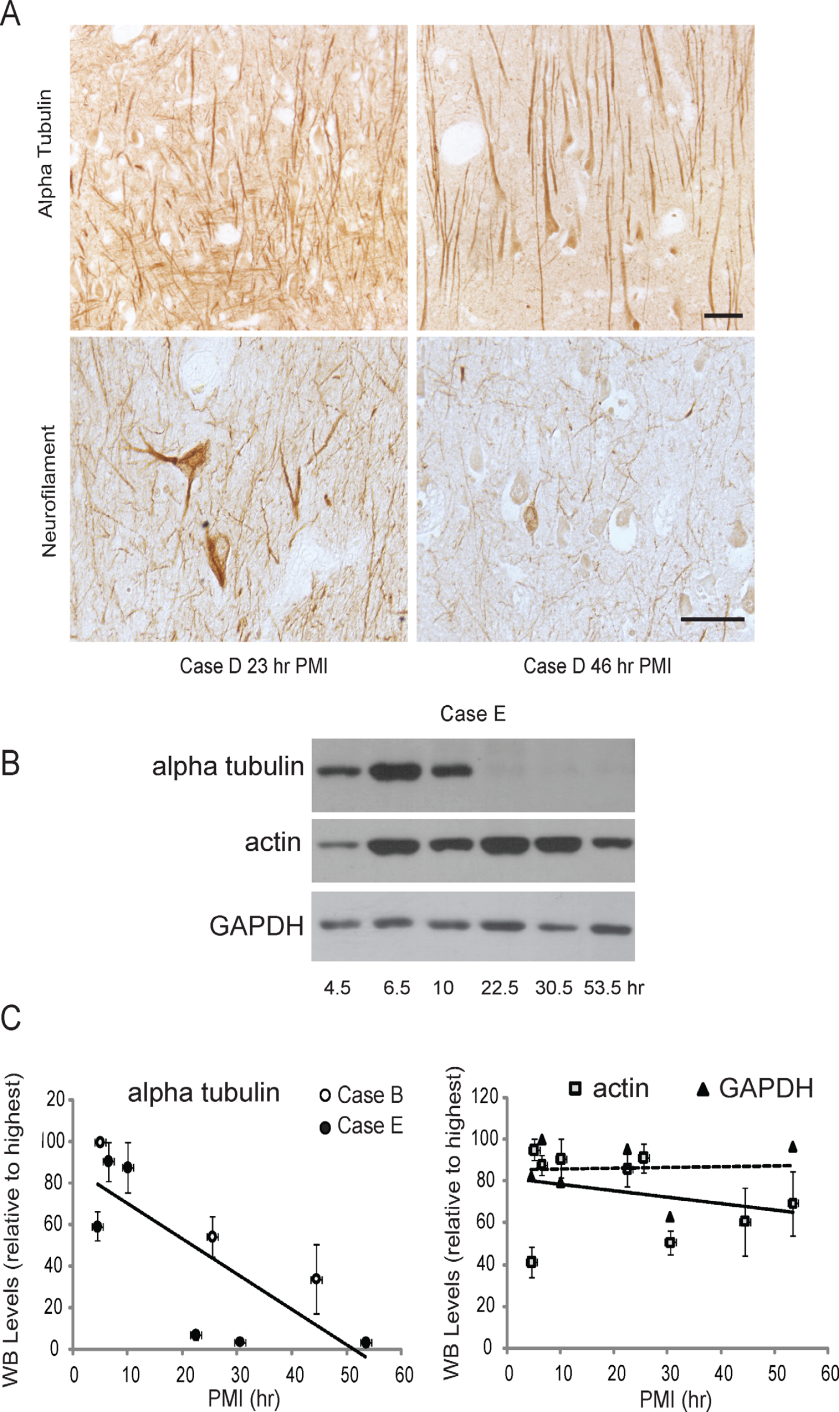
Effect of PMI on cytoskeletal proteins. (A) Neuronal cell bodies and processes are immunostained using antibodies to alpha tubulin and neurofilament proteins even at longer PMI times. Scale bars = 50 μm. (B) However, by Western blot, while the proteins actin and GAPDH show no correlation with PMI times, detectable tubulin levels decrease significantly after a PMI of 22 h. (C) Relative levels were based on the highest reactive band per case. In the graph showing tubulin quantification, both Case E (black circles) and Case B (white circles, show significant loss of tubulin Western blot reactivity with PMI time (p<0.02 using regression analysis. Quantification found no change with PMI time for either actin or GAPDH. Error bars represent SEM of three separate experiments.

Axonal processes of neurons remained immunoreactive for α-tubulin after 48 h in 3/6 cases (Fig 2A), and neurofilament proteins were still readily detectable with extended PMI, though some loss of intensity is apparent (Fig 2A). By Western blot, actin and GAPDH levels did not decrease even at the 48 h time point, however, tubulin levels dramatically decreased after 22 h (Fig 2B). Relative protein levels were based on the highest value for each case, and a regression analysis was used to determine that actin and GAPDH were not significantly lower with increased PMI, but tubulin levels showed a significant inverse correlation with increased PMI (Fig 2C; p<0.02).

### Effect of PMI on mitochondria and lipid peroxidation

**Fig 3:**
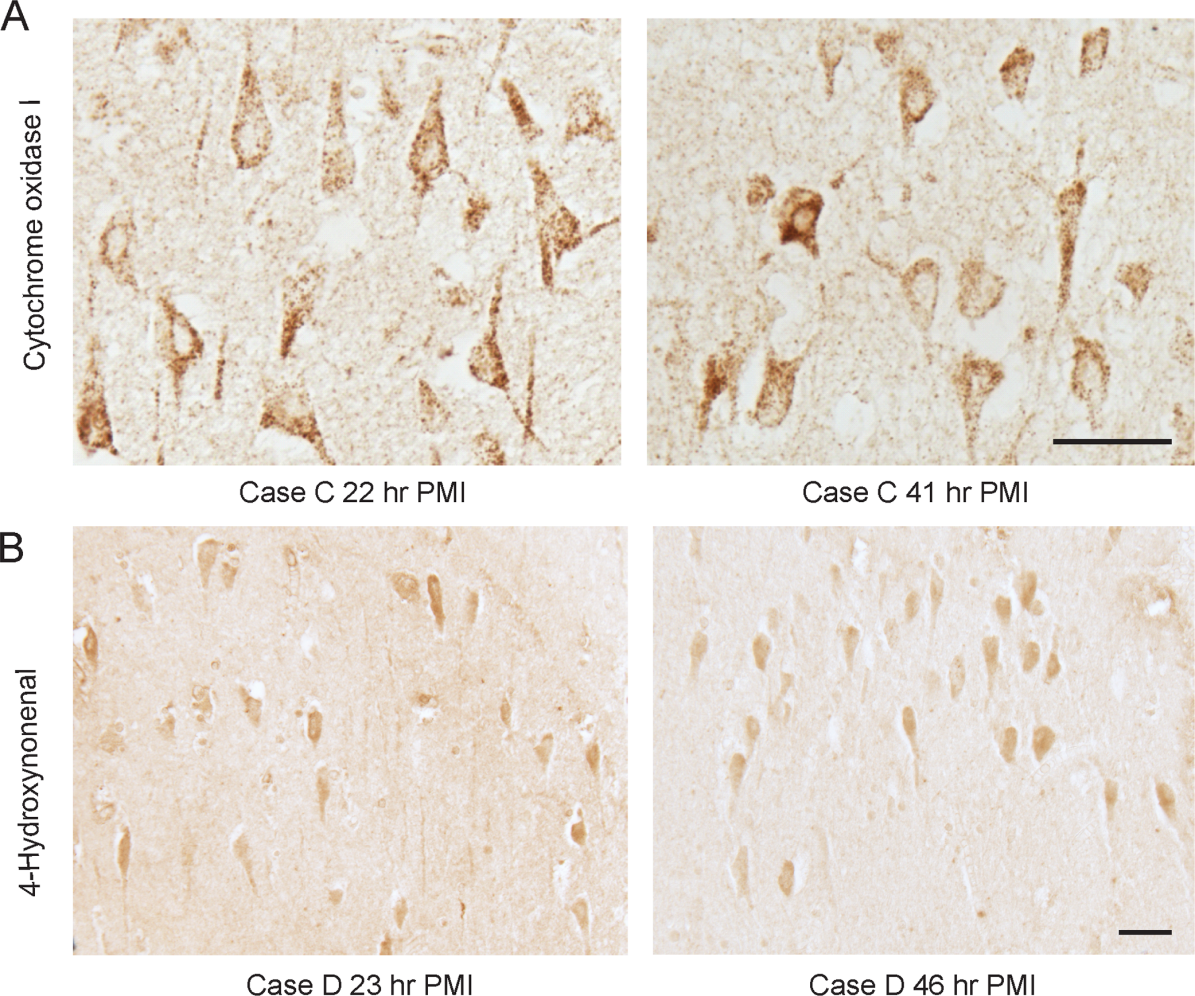
Effect of PMI on mitochondria and lipid peroxidation. Neither mitochondria morphology nor COX-1 immunoreactivity are affected by even the longest PMI studied. Lipid peroxidation demonstrated by an antibody to 4-hydroxynonenal also remains unchanged with time. Scale bars = 50 μm.

Immunohistochemistry with an antibody to COX-1, a mitochondrial protein, showed no loss of immunoreactivity in any case even after 48 h (Fig 3). The typical pattern and neuronal levels of COX-1 remained unchanged following extended PMI. Further, the lipid peroxidation product 4-hydroxynonenal was also present at similar levels in neurons at different PMIs.

### Effect of PMI on tau phosphorylation

**Fig 4:**
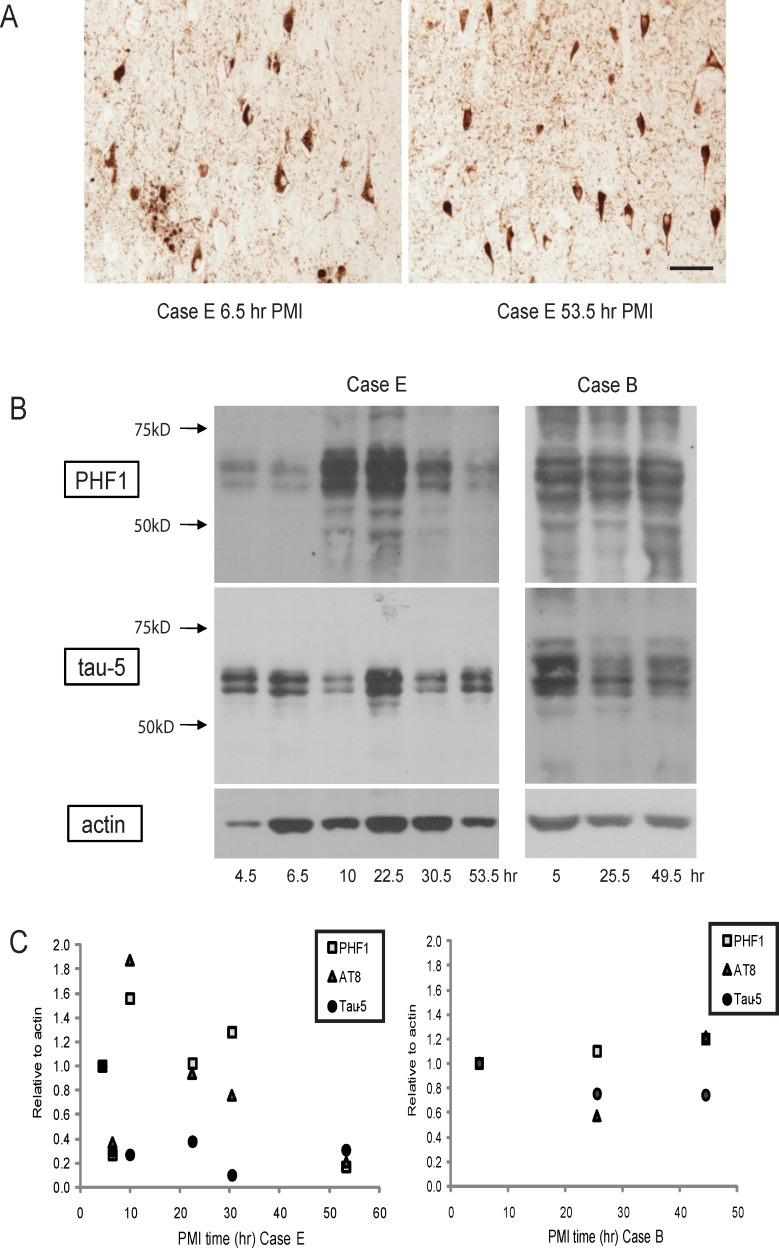
Effect of PMI on tau phosphorylation. (A) Neurofibrillary tangles are well stained by the phosphorylated tau antibody PHF1 in a case at both low PMI (6.5 h) and extended PMI (53.5 h). (B) Phosphorylated tau levels did not seem to correlate with increased PMI by Western blot (PHF1, AT8- not shown), but rather were variable in Case E (60 s exposure time shown) and similar in Case B (only 1 s exposure time from same blot). (C) Quantification revealed no trend of PMI with either PHF1, AT8 or tau 5 levels in either Case E or Case B.

For this study, we chose to examine phosphorylated tau proteins that are relevant to neurodegenerative disease research. Phosphorylated tau is a major component of the neurofibrillary pathology associated with Alzheimer disease. Even after a PMI of 53 h, neurofibrillary tangles were still strongly and clearly immunoreactive for phosphorylated tau (Fig 4A). By Western blot, primary antibodies PHF1, tau-5, and AT8 (not shown) detected bands at all PMI time points (Fig 4B). Case B had higher levels of phosphorylated tau compared to Case E, which required much longer exposure time compared to Case B to reveal the tau bands on the same blot. Also, while Case B showed little change in PHF1 reactivity with PMI time, Case E displayed variable levels that do not correlate with tau-5, actin, or PMI (Fig 4C). These results instead may reflect different levels of neurodegeneration and neuronal population sampling across the hippocampus.

### Effect of PMI on other commonly examined proteins

**Fig 5:**
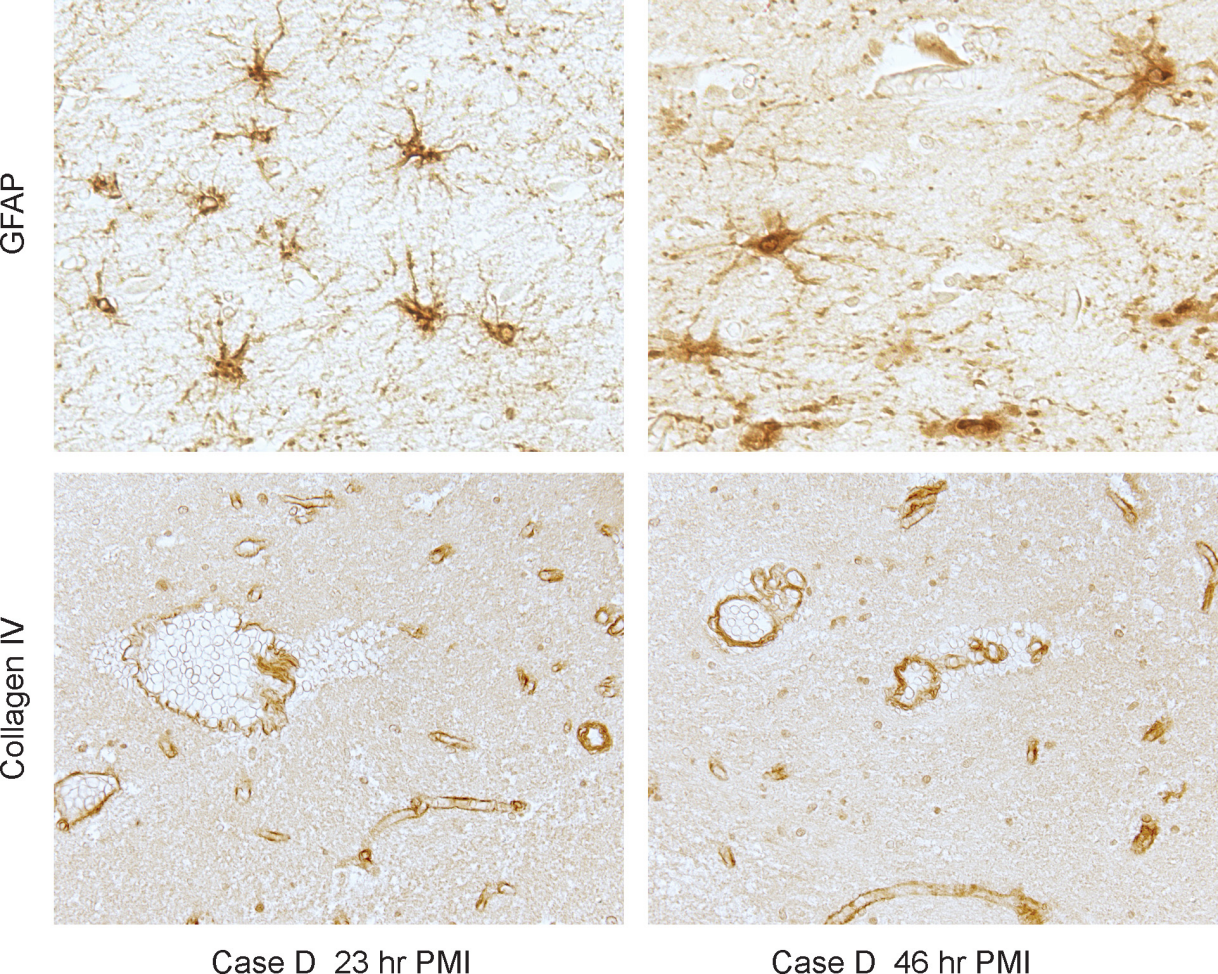
Effect of PMI on GFAP and collagen. Astrocytes also maintain their morphology and immunoreactivity for the protein GFAP even at long PMI, and brain vasculature shows no loss of structure or detectable levels of collagen.

GFAP is one of the common markers for astrocytosis. Immunocytochemistry with a GFAP antibody did not show significant changes in astrocyte staining with increased PMI (Fig 5). In addition, an antibody to collagen IV showed equivalent staining intensity of vessels even at the longest PMI (Fig 5).

### Effect of PMI on nucleic acids

**Fig 6:**
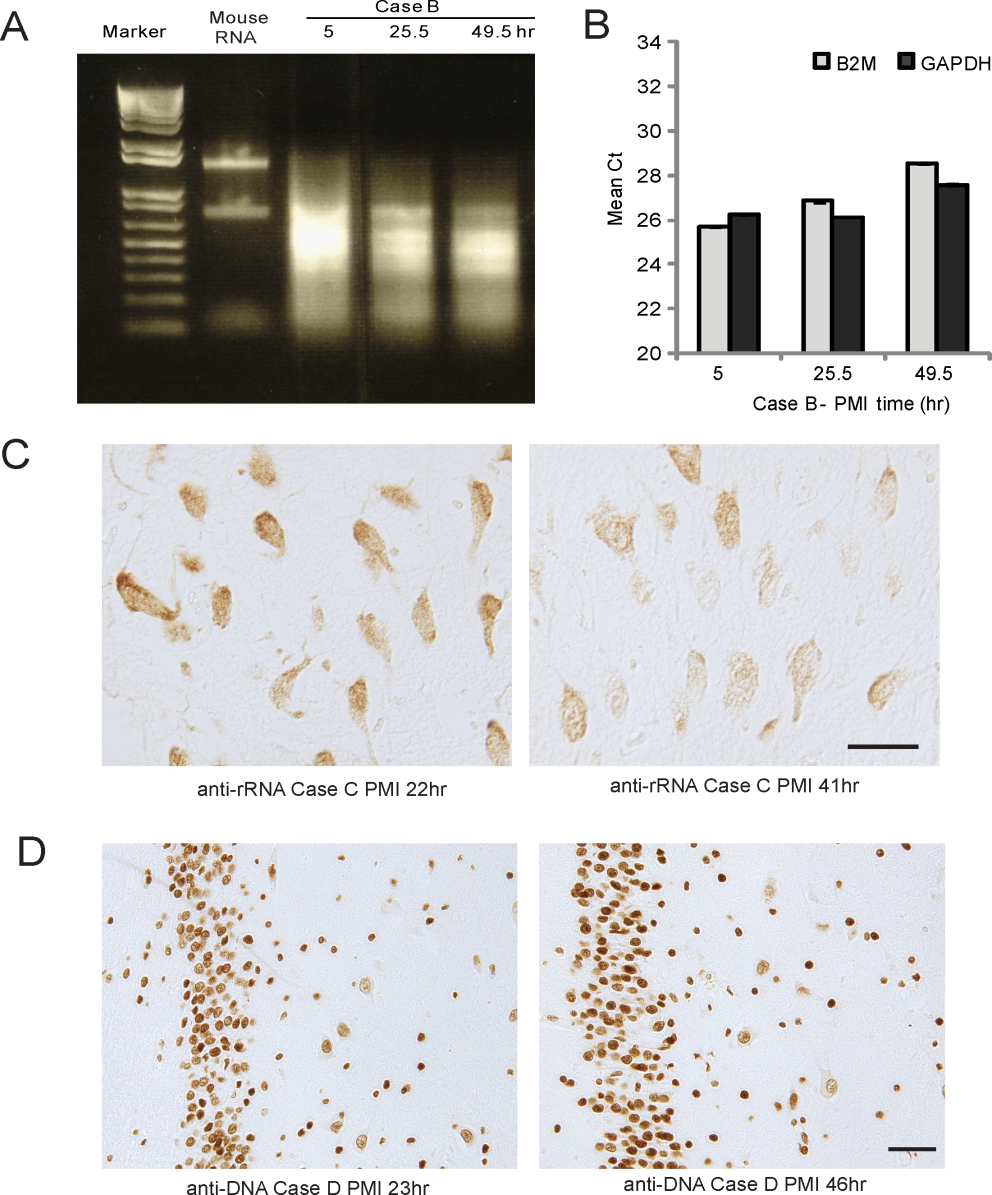
RNA degrades with time. (A) The ethidium bromide stained gel of Case B shows some RNA degradation with time. (B) qRT-PCR results show that there is increased Mean Ct with increasing PMI, yet short fragments of both GAPDH and B2M were amplified, even after 49.5 h for Case B. (C) An antibody to rRNA shows a similar decrease of immunoreactivity with time, (D) but an antibody to dsDNA remains robust even after a PMI of 46 h. Scale bars = 50 μm.

RNA was extracted from the frozen tissue samples collected from Cases B and E. Fig 6A shows the ethidium bromide stained gel of the purified RNA with increasing PMI from Case B. While a significant amount of RNA was obtained after 49.5 h, even at the shortest PMI of 5 h no intact RNA is observed, when compared to mouse RNA. cDNA was successfully produced from these RNA samples and common housekeeping genes, GAPDH and B2M, were successfully detected by qRT-PCR, indicating that significantly degraded RNA can generate short products in qRT-PCR analysis (Fig 6B). Case B showed slight increases in Mean Ct with increased PMI for B2M and GAPDH, reflecting the RNA degradation seen in the gel. However, for Case E, RNA yields from samples collected at the later time points, 30.5 and 53.5 h PMI, were too low to perform qRT-PCR, even after multiple attempts at RNA purification. Overall, higher Mean Ct were observed (greater than 30) confirming the overall RNA degradation seen in the gel in which only smears and no defined RNA bands were seen (data not shown).

Morphologically, an antibody specific to ribosomal RNA (rRNA) corroborates the qRT-PCR results such that in all cases, rRNA immunostaining intensity was decreased with time. Fig 6C shows hippocampal neurons from Case C tissue fixed at 22 h and 41 h PMI. Conversely, the number and intensity of nuclei stained using an antibody to DNA remained unchanged even after 46 h at 4°C (Fig 6D).

## Discussion

Experiments about PMI all have limitations, whether it is comparing different cases or storage conditions, having to use various tissue regions, or even animal models that do not mimic the day-to-day autopsy setting. This study characterized the effect of PMI within the same patient tissues, carefully controlling for brain region analyzed and tissue handling, yet choosing a random selection of autopsy cases and examining proteins commonly used for diagnosis and in research studies with the goal of determining the effects of extended PMI. Even so, the temperature of the brain tissue at time of autopsy was not measured here and may be more relevant than PMI, since it may not correlate with PMI time due to the many other confounding factors. Indeed, a classic study found that it takes 30 h for the human brain to cool when the body is refrigerated at 8°C [12].

One finding from the present study is that individual patient samples are very different even when collected, stored, and handled under the same conditions and by the same personnel. Perhaps other factors including cause of death, aging, disease, medications, other medical interventions, presence of fever or hypothermia, and overall health at the time of death may affect tissue preservation [13]. For instance, RNA integrity was very different in the two cases analyzed. Even though these two cases had initial PMI of 4 h, and then were sampled at additional time points, one case (Case B) had greater RNA preservation compared to the other case (Case E). Indeed, in the samples from Case B stored for up to 49.5 h at 4°C, RNA for commonly analyzed housekeeping genes, GAPDH and B2M, had sufficiently long fragments to be measured by qRT-PCR, while the RNA samples from Case E were more extensively degraded even at the earliest PMI time. These results suggest that RNA degradation is gradually increased by PMI, but the initial state of the RNA is more critical for further RNA analysis. This is in agreement with previous literature that shows a bias in trend lines with a PMI of more than 9 h for some transcripts, but no change in the housekeeping gene β-actin transcripts [14]. Our results are in agreement with other studies that conclude that PMI has little effect on RNA and that antemortem factors may be a greater influence [15]. Further, nuclear integrity was maintained in all cases even after 48 h of storage prior to fixation, since DNA immunostaining revealed strong and very specific nuclear reactivity, without any loss of nuclear morphology. Other studies have shown that DNA repair activity is also maintained up to a PMI of 24 h in rodent model [16]. Conclusions from these studies are that while PMI indeed affects RNA integrity collected from a specific patient sample and should be a consideration for human studies using archived tissue samples, the variability between individual patients is more apparent. Indeed, the variation of NeuN immunostaining and immunblotting between the two cases shown in Fig 1 likely represent individual patient variability and disease state, and not PMI, as has been suggested previously [17].

Another important finding of this study is that the proteins actin and GAPDH, often used as internal loading controls for Western blot studies, remain intact even at the longest PMIs studied- over 48 h. These data corroborate studies performed in cohorts of rodents with different PMIs showing that actin remains stable until the 48 h time point [7]. Conversely, slight overall protein degradation as detected by Coomassie blue at a PMI of 24 h agrees with rodent studies [7, 18]. Importantly, another common internal loading control, tubulin, decreases with time; by 22 h PMI more than 50% of the tubulin is lost by Western blot analysis. This observation was confirmed in two separate cases. Indeed, in one case, tubulin levels dropped by 90%. However, even with a PMI of 48 h, neuronal processes were still evident in the fixed tissue samples as demonstrated by the strong and specific tubulin immunoreactivity found in many, but not all cases. We previously reported that tubulin modifications are prevalent and well maintained in neurons and by Western blot in cases with PMI less than 10 h [19]. In the present study we found that majority of tubulin loss occurs after a PMI of 12 h. Historically, the ability to detect tubulin prepared from human brain has been correlated with PMI [20] and our study provides further evidence that for some proteins, with increased PMI time, enough protein degradation occurs to cause significant loss of detectable protein during the preparation of tissue homogenization, lysis buffer extraction, and electrophoresis.

Other commonly used markers remain unchanged following a PMI of 24 or even 48 h. For instance, GFAP, a marker protein for astrocytes, is unaffected by increased PMI. COX-1, a subunit of complex IV made in the mitochondria, also remains detectable, which is in direct support of a study that found mitochondria morphology and even activity is maintained with a PMI up to 24 h in an experimental model [21]. The phosphorylated tau-containing neurofibrillary tangles maintain their structure and protein integrity, as well. Variable levels of tau detected by PHF1 and AT8 were seen in one case by Western blot; this was likely related to changes in pathology accumulation across the sample rather than PMI. A mouse expressing mutant tau has been shown to exhibit PMI effects on tau-5 staining, however the species difference or presence of the P301L mutation may explain this apparent discrepancy [18]. Others have shown that there is a significant loss of tau phosphorylation within minutes in both human biopsy samples [10] and in mice [22], and that this loss is exacerbated by not chilling immediately on ice, as substantial dephosphorylation occurs within the short time required for perfusion. Based on these studies, we can only assume that even at our earliest PMI of 4 h we are detecting diminished levels of tau phosphorylation to begin with, and indeed our findings agree with Matsuo et al. [10] that dephosphorylation slows after time likely due to the diminution of the activity of phosphatases including PP2A. Overall tissue morphology is maintained such that neuronal structures and the vasculature are not disrupted.

## Conclusion

In many studies using human autopsy tissues, PMI is often reported as a patient variable. This study confirms that PMI is indeed a valuable parameter to report, and care must be taken when examining certain proteins and nucleic acids. Importantly, even though we found that actin or GAPDH levels remained unchanged with PMI, it should not be the only factor used to control for total protein, since in the same cases loss of detectable tubulin was significant after 22 h. However, for all proteins analyzed here, PMIs <22 h exhibited virtually no loss of protein integrity by Western blot. Thus, in studies using archival frozen tissue samples, levels of target proteins should also be correlated to PMI to ensure that any changes observed are not a direct result of tissue handling. Using immunohistochemical methods, however, all proteins examined in this study were still detected in most cases and without any substantial morphological change when fixed even after a 48 h PMI. These results, together with previous reports using biopsy samples and animal models, suggest that temperature of the tissue may be more relevant for biochemical studies, and may be another worthy factor to measure when tissue sampling is to be done proactively. The findings reported in this study are critical for basic research studies and emphasize the importance of having sufficient numbers of cases to overcome the variability inherent when using human samples.

## References

[1] Chandana R, Mythri RB, Mahadevan A, Shankar SK, Srinivas Bharath MM. Biochemical analysis of protein stability in human brain collected at different post-mortem intervals. Indian J Med Res. 2009;129(2):189–99. . 19293447

[2] Kolasinski J, Stagg CJ, Chance SA, Deluca GC, Esiri MM, Chang EH, et al A combined post-mortem magnetic resonance imaging and quantitative histological study of multiple sclerosis pathology. Brain. 2012;135(Pt 10):2938–51. 10.1093/brain/aws242 23065787 PMC3470716

[3] Kim TH, Zollinger L, Shi XF, Rose J, Jeong EK. Diffusion tensor imaging of ex vivo cervical spinal cord specimens: the immediate and long-term effects of fixation on diffusivity. Anat Rec (Hoboken). 2009;292(2):234–41. 10.1002/ar.20823 19051255 PMC2860544

[4] Tomita H, Vawter MP, Walsh DM, Evans SJ, Choudary PV, Li J, et al Effect of agonal and postmortem factors on gene expression profile: quality control in microarray analyses of postmortem human brain. Biol Psychiatry. 2004;55(4):346–52. 10.1016/j.biopsych.2003.10.013 14960286 PMC3098566

[5] Dressler J, Hanisch U, Kuhlisch E, Geiger KD. Neuronal and glial apoptosis in human traumatic brain injury. Int J Legal Med. 2007;121(5):365–75. 10.1007/s00414-006-0126-6 . 17106737

[6] Monoranu CM, Apfelbacher M, Grunblatt E, Puppe B, Alafuzoff I, Ferrer I, et al pH measurement as quality control on human post mortem brain tissue: a study of the BrainNet Europe consortium. Neuropathol Appl Neurobiol. 2009;35(3):329–37. 10.1111/j.1365-2990.2008.01003a.x 19473297 PMC4532399

[7] Halim ND, Weickert CS, McClintock BW, Hyde TM, Weinberger DR, Kleinman JE, et al Presynaptic proteins in the prefrontal cortex of patients with schizophrenia and rats with abnormal prefrontal development. Mol Psychiatry. 2003;8(9):797–810. 10.1038/sj.mp.4001319 . 12931207

[8] Li J, Gould TD, Yuan P, Manji HK, Chen G. Post-mortem interval effects on the phosphorylation of signaling proteins. Neuropsychopharmacology. 2003;28(6):1017–25. 10.1038/sj.npp.1300112 . 12637955

[9] Li X, Friedman AB, Roh MS, Jope RS. Anesthesia and post-mortem interval profoundly influence the regulatory serine phosphorylation of glycogen synthase kinase-3 in mouse brain. J Neurochem. 2005;92(3):701–4. 10.1111/j.1471-4159.2004.02898.x 15659239 PMC1850892

[10] Matsuo ES, Shin RW, Billingsley ML, Van deVoorde A, O'Connor M, Trojanowski JQ, et al Biopsy-derived adult human brain tau is phosphorylated at many of the same sites as Alzheimer's disease paired helical filament tau. Neuron. 1994;13(4):989–1002. . 7946342 10.1016/0896-6273(94)90264-x

[11] Williams WM, Torres S, Siedlak SL, Castellani RJ, Perry G, Smith MA, et al Antimicrobial peptide beta-defensin-1 expression is upregulated in Alzheimer's brain. J Neuroinflammation. 2013;10:127 10.1186/1742-2094-10-127 24139179 PMC3817866

[12] Perry RH, Tomlinson BE, Taylor MJ, Perry EK. Human brain temperature at necropsy: a guide in post-mortem biochemistry. Lancet. 1977;1(8001):38 . 63669 10.1016/s0140-6736(77)91669-5

[13] Hynd MR, Lewohl JM, Scott HL, Dodd PR. Biochemical and molecular studies using human autopsy brain tissue. J Neurochem. 2003;85(3):543–62. . 12694381 10.1046/j.1471-4159.2003.01747.x

[14] Popova T, Mennerich D, Weith A, Quast K. Effect of RNA quality on transcript intensity levels in microarray analysis of human post-mortem brain tissues. BMC Genomics. 2008;9:91 10.1186/1471-2164-9-91 18298816 PMC2268927

[15] Gutala RV, Reddy PH. The use of real-time PCR analysis in a gene expression study of Alzheimer's disease post-mortem brains. J Neurosci Methods. 2004;132(1):101–7. . 14687679 10.1016/j.jneumeth.2003.09.005

[16] Soltys DT, Pereira CP, Ishibe GN, de Souza-Pinto NC. Effects of post mortem interval and gender in DNA base excision repair activities in rat brains. Mutat Res. 2015;776:48–53. 10.1016/j.mrfmmm.2015.01.003 . 26255940

[17] Unal-Cevik I, Kilinc M, Gursoy-Ozdemir Y, Gurer G, Dalkara T. Loss of NeuN immunoreactivity after cerebral ischemia does not indicate neuronal cell loss: a cautionary note. Brain Res. 2004;1015(1–2):169–74. 10.1016/j.brainres.2004.04.032 . 15223381

[18] Sahara N, Murayama M, Mizoroki T, Urushitani M, Imai Y, Takahashi R, et al In vivo evidence of CHIP up-regulation attenuating tau aggregation. J Neurochem. 2005;94(5):1254–63. 10.1111/j.1471-4159.2005.03272.x . 16111477

[19] Zhang F, Su B, Wang C, Siedlak SL, Mondragon-Rodriguez S, Lee HG, et al Posttranslational modifications of alpha-tubulin in Alzheimer disease. Transl Neurodegener. 2015;4:9 10.1186/s40035-015-0030-4 26029362 PMC4448339

[20] Kosik KS, Gilbert JM, Selkoe DJ, Strocchi P. Characterization of postmortem human brain proteins by two-dimensional gel electrophoresis. J Neurochem. 1982;39(6):1529–38. . 7142987 10.1111/j.1471-4159.1982.tb07985.x

[21] Barksdale KA, Perez-Costas E, Gandy JC, Melendez-Ferro M, Roberts RC, Bijur GN. Mitochondrial viability in mouse and human postmortem brain. FASEB J. 2010;24(9):3590–9. 10.1096/fj.09-152108 20466876 PMC2923351

[22] Wang Y, Zhang Y, Hu W, Xie S, Gong CX, Iqbal K, et al Rapid alteration of protein phosphorylation during postmortem: implication in the study of protein phosphorylation. Sci Rep. 2015;5:15709 10.1038/srep15709 26511732 PMC4625177

