# Correction: Individual Case Analysis of Postmortem Interval Time on Brain Tissue Preservation

**DOI:** 10.1371/journal.pone.0157209

**Published:** 2016-06-03

**Authors:** Jeffrey A. Blair, Chunyu Wang, Damarys Hernandez, Sandra L. Siedlak, Mark S. Rodgers, Rohan K. Achar, Lara M. Fahmy, Sandy L. Torres, Robert B. Petersen, Xiongwei Zhu, Gemma Casadesus, Hyoung-gon Lee

The sixth author’s first name is spelled incorrectly. The correct name is: Rohan K. Achar. The correct citation is: Blair JA, Wang C, Hernandez D, Siedlak SL, Rodgers MS, Achar RK, et al. (2016) Individual Case Analysis of Postmortem Interval Time on Brain Tissue Preservation. PLoS ONE 11(3): e0151615. doi:10.1371/journal.pone.0151615.
